# On a New Species of Torticollis

**Published:** 1841-05

**Authors:** 


*On a New Species of Torticollis.* By M. Bouvier.—This species of torticollis differs altogether from the acute or chronic muscular form, and from the spontaneous luxation of the atlas and axis, three states with which it has been confounded. It has its seat in the articulations of the first cervical vertebræ, and may perhaps be designated by the term articular torticollis. The lateral articulations, right or left, of the first cervical vertebræ, and especially of the atlas with the axis, are, so to speak, the focus of articular torticollis, which consists in a peculiar form of inflammation of the synovial capsule and fibrous tissues of these articulations. The essential characters are: 1. A position of the head and neck, similar to that produced by contraction of one of the sterno-cleido-mastoid muscles, namely, lateral flexion to the right or left, and rotation of the face to the opposite side. 2. A pain felt towards the top and sides of the nape of the neck, sometimes on the lateral and posterior parts of the cranium; arising spontaneously, or in the movements of the neck. 3. Complete relaxation of the sterno-cleido-mastoid of the affected side, and an equal tension of the muscles of the neck both left and right. 4. Stiffness and pain in all or any of its movements, notwithstanding the preservation of the articular connexions of the cervical vertebræ, and the integrity of the muscles. 5. Absence of swelling, and of sensibility on pressure. 6. Atrophy, or arrest of development of the side of the face, which answers to the inclination of the head, when this has lasted a certain time.

This disease presents two successive states or periods, the acute and chronic. It is often produced by cold, sometimes by sudden distension of the ligaments. M. Bouvier thinks it very important to detect this disease, as the section of the muscle would be useless and rash, but reserves to another occasion the proceedings which he has found useful.—*Brit. and For. Med. Rev., from l'Experience.* 17 *Septembre,* 1840. *Med. Exam., Feb.* 27, 1841.

